# A highly efficient method to differentiate CGRP-expressing peptidergic nociceptors from human induced pluripotent stem cells

**DOI:** 10.1016/j.stemcr.2026.102971

**Published:** 2026-06-25

**Authors:** Galbha Duggal, Xinyu Li, Philippa Pettingill, Tatjana Lalic, Shailesh Kumar Gupta, Christine Flodgaard Høgsbro, Viola Volpato, Caleb Webber, Rory Bowden, Despoina Charou, Kanisa Arunasalam, Marcello Maresca, Ryan Hicks, Satyan Chintawar, M. Zameel Cader

**Affiliations:** 1 Nuffield Department of Clinical Neurosciences, University of Oxford, Oxford, UK; 2 Oxford StemTech, The Oxford Science Park, Oxford, UK; 3 Global Portfolio & Project Management, Oncology R&D, Cambridge, UK; 4 Department of Neurology, Faculty of Health and Medical Sciences, Danish Headache Center, Glostrup Hospital, University of Copenhagen, Glostrup, Denmark; 5 UK Dementia Research Institute, Department of Psychological Medicine and Clinical Neuroscience, Cardiff University, Cardiff, UK; 6 Wellcome Trust Centre for Human Genetics, University of Oxford, Oxford, UK; 7 Discovery Biology, Discovery Sciences IMED Biotech Unit, AstraZeneca, Gothenburg, Sweden; 8 Bioanalytical and Biomarker Labs, Thermofisher Scientific, Gothenburg, Sweden

**Keywords:** iPSC, nociceptor, pain, sensory neurons, CGRP, peptidergic, trigeminal, migraine, human, inflammation

## Abstract

Pain disorders such as neuropathic pain and headache remain areas of considerable unmet need and considered high risk by pharma. Human-induced pluripotent stem cells (iPSC)-derived sensory neurons have already been used to accelerate translational research but the current differentiation protocols produce non-peptidergic nociceptors. We demonstrate for the first time the robust differentiation of hiPSC into peptidergic nociceptor lineage with high yield. These nociceptors express CGRP and TRPV1 and show functional maturity including the expression of TTX-resistant currents and responding to TRPV1 and TRPA1 agonists. Importantly, they were able to release CGRP basally and upon stimulation by inflammatory soup, which was inhibited upon the application of the 5-HT_1B/1D/1F_ agonist, sumatriptan, a migraine prophylactic drug. We report the successful generation of a novel *in vitro* functional peptidergic nociceptor model which will allow investigation of disease mechanisms in pain and translational phenotypic drug screening for new effective pain therapies.

## Highlights

• Differentiation of human iPSCs into CGRP-releasing peptidergic nociceptors
• Functional maturity demonstrated by TTX-resistant currents and TRPV1/TRPA1 responses
• Basal and inflammatory-evoked CGRP release is inhibited by sumatriptan and topiramate
• Provides a translational *in vitro* platform for pain mechanism studies and drug discovery

## Teaser

Cader and colleagues report the first robust generation of human iPSC-derived peptidergic nociceptors with functional maturity and stimulus-evoked CGRP release. This high-yield, translationally relevant model overcomes limitations of existing protocols and provides a powerful platform to study pain mechanisms and accelerate the development of new therapeutics for migraine and neuropathic pain.

## Introduction

Sensory neurons are peripheral afferent nerve endings of specialized stimulus-detecting modalities: nociceptors for pain, mechanoreceptors for pressure and touch, and proprioceptors for the movement and position of body parts. The soma of sensory neurons resides in dorsal root ganglia (DRG) and trigeminal ganglia (TG), with long processes extending toward both the periphery and the central nervous system for signal relay. Stratification of sensory neurons is based on axonal biophysical properties, the extent of myelination, and selective marker expressions. Nociceptors are the detectors and initiators of pain signals, consisting of thinly myelinated A_δ_-fibers and unmyelinated C-fibers. Conventionally, small diameter C-fibers are further categorized by differential developmental trajectories and ion channel expression. Peptidergic nociceptors are characterized by the expression of neuropeptides including calcitonin gene-related peptide (CGRP), Substance P, and pituitary adenylate cyclase activating polypeptide-38 (PACAP-38) and transient receptor potential (TRP) cation channels such as heat sensor subfamily V member 1 (TRPV1) and extreme cold sensor subfamily A member 1 (TRPA1) (Edvinsson et al., 2018; Liu and Ma, 2011a; Wainger et al., 2015). Non-peptidergic nociceptors typically express purinergic receptor P2RX3 and cold sensory TRP channel subfamily M member 8 (TRPM8) (Liu and Ma, 2011a).

TG and DRG neurons are usually assumed to be similar, despite different cellular compositions, differential transcriptomic profiles, and distinct innervation destination (Megat et al., 2019). Peptidergic nociceptors, especially of TG origins, are of considerable interest in disorders such as migraine as they regulate pain, modulate peripheral and cerebral blood flow, and initiate or sustain inflammatory responses (Benemei et al., 2017; Edvinsson and Warfvinge, 2019; Lukacs et al., 2017). CGRP secreted from trigeminal peptidergic nociceptors has been demonstrated to trigger neurogenic inflammation and vasodilation in migraine pathophysiology during an attack (Russell et al., 2014). CGRP receptor antagonists (Bell, 2014) and more recently CGRP monoclonal antibodies (Goadsby et al., 2017; Oliveira et al., 2024; Silberstein et al., 2017) are showing good efficacy in migraine clinical trials, with the first migraine preventative drug recently approved by the U.S. Food and Drug Administration (Edvinsson et al., 2018).

As most developmental studies for nociceptor cell fate specification are conducted in rodents or chick embryos, understanding of human nociceptor development is limited, especially for the peptidergic pathway. Recent development of transcriptomic profiling techniques has enabled the detailed examination of gene expression profiles in human nociceptors at the single-cell level, thereby permitting interspecies comparisons. Although the major clusters of nociceptors remain conserved in human and mice, most human nociceptors display some aspects of the conventional peptidergic phenotype as broad expression of NaV1.8, NaV1.9, TRPV1, Substance P, and CGRP. This is unique in human nociceptors compared to rodents (Jung et al., 2023; Lu et al., 2024; Middleton et al., 2021; Nguyen et al., 2021; Ray et al., 2018; Rostock et al., 2018; Shiers et al., 2020, 2021; Tavares-Ferreira et al., 2022; Yang et al., 2022). There are also significantly more nociceptors in human with co-expression of TRPV1/P2RX3 and CGRP/P2RX3 compared to rodent counterparts (Shiers et al., 2020, 2021).

Nevertheless, it has been possible to chemically derive functional nociceptors *in vitro* from pluripotent stem cells including human embryonic stem cells (hESCs) and human-induced pluripotent stem cells (hiPSCs) which expressed RUNX1 and responded to ATP (Chambers et al., 2012, Pettingill et al., 2019). The neurons yielded from the Chambers’ protocol accurately reciprocated the convergent expression of TRPV1 and P2RX3. However, the lack of electrophysiologically active TRPV1 to selective agonist capsaicin or neuropeptide secretion distinguishes Chambers’ nociceptors as non-peptidergic of a DRG origin (Röderer et al., 2023; Schoepf et al., 2020; Young et al., 2014).

Successive attempts on inducing peptidergic cell fates focussed on improving TRPV1 responsiveness, either via chemical induction (Deng et al., 2023; Guimarães et al., 2018; Muller et al., 2018; Wilson et al., 2018), ectopic expression of key transcription factors (Blanchard et al., 2015; Wainger et al., 2015), or a combination of both (Hulme et al., 2020; Lee et al., 2015; Schrenk-Siemens et al., 2022; Vojnits et al., 2019). Starting materials were not limited to pluripotent stem cells, and also included fibroblasts, epidermal neural crest stem cells, or blood-induced neural progenitor cells. However, only a few studies tested for secretive neuropeptides, e.g., substance P (Deng et al., 2023; Guimarães et al., 2018; Muller et al., 2018), and CGRP (Muller et al., 2018) via ELISA. Although promising CGRP release was shown in murine fibroblast-derived nociceptors in one study, the overall yield of peptidergic neurons was low and nociceptors derived from human fibroblasts were not investigated for CGRP expression and release (Wainger et al., 2015). All recent attempts also primarily induced neural crest progenitors that would derive DRG nociceptors. Alternatively, a modified dual-SMAD method (Dincer et al., 2013) drives early nociceptors progenitors into cranial placode derivatives to generate TG nociceptors instead.

As the current established protocols for nociceptor differentiation recapitulate only the neural crest non-peptidergic fate *in vitro*, we aimed to formulate an optimized protocol using hiPSCs to induce the peptidergic nociceptive lineage. Here, we demonstrate a method which can be adapted to produce either non-peptidergic TRPA1-expressing nociceptors (∼45%) which has not been shown previously or TRPV1/CGRP expressing peptidergic nociceptors (∼93%). The peptidergic nociceptors were able to release CGRP and responded to nociceptive stimuli including capsaicin and inflammatory soup or efficacious migraine therapies. Remarkably, we show that by co-culture with astrocytes, *in vitro*-derived TG early nociceptors can switch their fate from a non-peptidergic to peptidergic nociceptor type. The highly efficient production of peptidergic nociceptors *in vitro* from hiPSCs opens new avenues in drug-disease modeling thereby enhancing the possibilities of successful therapies for pain.

## Results

### Optimization and temporal molecular profiling of human iPSC-derived TG nociceptors for peptidergic and non-peptidergic nociceptive markers

**Figure 1:**
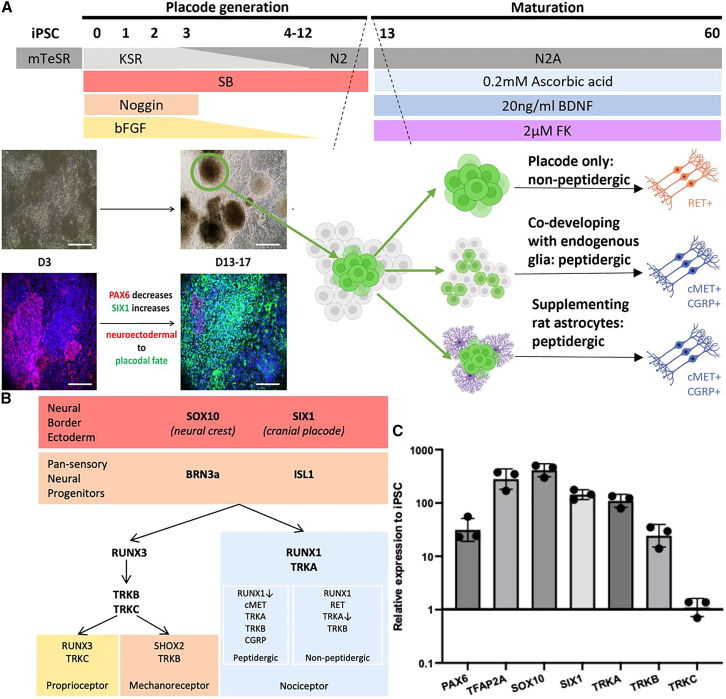
Differentiation of human iPSCs toward TG nociceptors (A) Schematic representation of modified TG nociceptor differentiation protocol. Human iPSCs are subjected to dual SMAD inhibition and either subjected to placodal isolation or no isolation or no isolation and replating strategies, and undergo maturation until day 60. Maturation methods lead to competitive cell fate specifications, highlighted by differential expressions of marker genes by immunofluorescence, scale bars, 25 μm. (B) Schematics of developmental trajectory of sensory neuron development based on rodent studies. (C) Expression of critical genes representing ectodermal lineages and sensory neuron subtypes by RT-qPCR at d12, *n* = 3 independent replicates.

To understand the *in vitro* specification of nociceptors toward a peptidergic or a non-peptidergic fate, we chemically induced trigeminal placodes as previously described (Dincer et al., 2013) and optimized placodal isolation and maturation (Figure 1A). Extended single SMAD inhibition successfully induced the expression of neural border markers SOX10 (neural crest) and SIX1 (cranial placode) with minimized expression and distribution of PAX6+ neuroectoderm (Figures 1C and 1D). Minimal *TRKC* expression was present, as the lack of proprioceptors is typical of TG in contrast to DRG, whereas significant upregulation of *TRKA* and *TRKB* were detected by quantitative reverse-transcription PCR (RT-qPCR) (Figure 1C).

Placodes could be identified as raised aggregates enriched of neural border progenitors (Figure 1A). As TG *in vivo* contain both neural border progenitors which are the ectodermal lineages that develop into sensory neurons, we manually isolated these placodes for maturation. Placodes maturing on PLO/LAM coating based on the original protocol were positive with bright red CASPASE-3 immunostaining, suggesting compromised cell viability (Figure S1A). Therefore, we replaced the basement membrane matrix with Matrigel for enhanced neuronal survival (Choi et al., 2014; Uemura et al., 2010) and supplemented pro-survival cyclic AMP agonist forskolin (Lepski et al., 2013; Li et al., 2000). Neurons cultured by the improved combination displayed mature electrophysiological functionalities with a healthy resting membrane potential of −60 mV. CASPASE-3 immunoreactivities were also lowered (Figure S1B).

**Figure 2:**
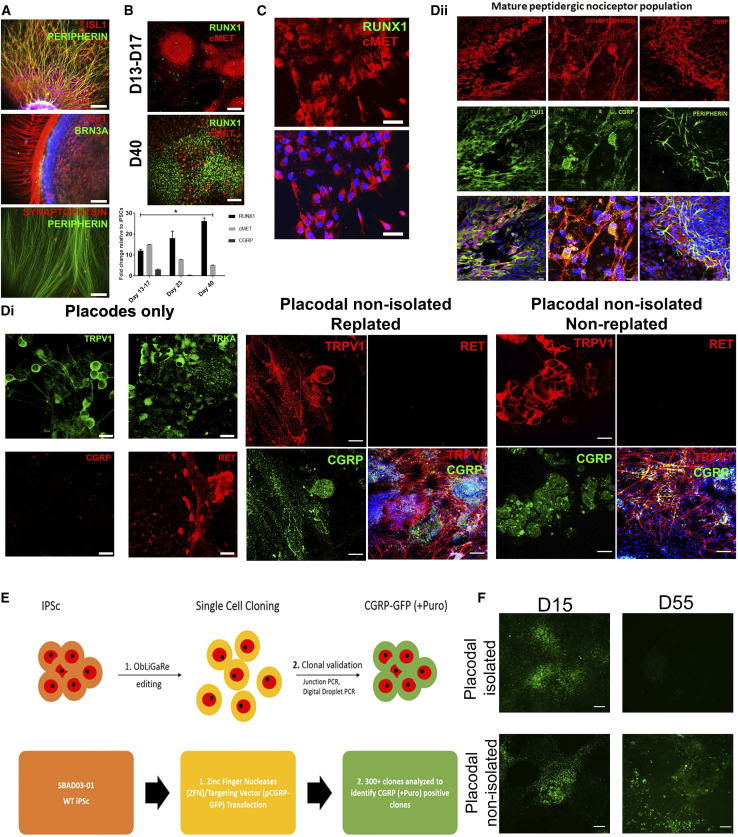
Presence of non-placodal cells re-directs neuronal cell fate specification, confirmed by CGRP expression (A) Confirmation of generic sensory neuron marker expression (ISL1/BRN3A, PERIPHERIN, TUJ1, SYNAPTOPHYSIN) of TG neurons, scale bars, 25 μm. (B) Time course of RUNX1 and cMET expression during development in TG non-peptidergic nociceptors from isolated placodes by immunofluorescence and RT-qPCR, scale bars, 25 μm, *n* = 3 independent replicates, respectively. (C) A small subset of neurons with retained cMET expression in TG non-peptidergic nociceptors from isolated placodes, scale bars, 50 μm. (D) (i) Comparison of terminal cell fates in RUNX1^+^/RET^+^/TRKA^+^/TRPV1^+^ non-peptidergic nociceptor subtypes by placodal isolation (left) and RUNX1-/CGRP+/TRPV1+ peptidergic subtypes by replating whole cultures (right) by immunofluorescence. Immunocytochemistry performed on CGRP-GFP reporter line with expression overlap of CGRP antibody, for whole replated cultures. (ii) TG peptidergic nociceptors derived upon non-placodal isolation replating strategy expressing nociceptive markers (ISL1, PERIPHERIN), neuronal marker TUJ1, synaptic marker SYNAPTOPHYSIN and peptidergic marker CGRP, scale bars, 25, 50, and 25 μm (L-R). (E) Workflow for generation of CGRP-GFP reporter line. (F) CGRP-GFP (reporter line) expressed in mature TG nociceptors in the absence of placodal isolation, with or without replating (bottom, scale bars, 5 μm).

From isolated placodes, we determined the time frame of commitment to non-peptidergic fates by temporally profiling the relative expression of non-peptidergic regulator RUNX1 and peptidergic hallmark cMET (Chen et al., 2006; Kobayashi et al., 2012). Expression of RUNX1 superseded that of cMET as maturation progressed, both at transcriptional and translational levels (Figure 2B). The relative abundance of *CGRP* transcript was extinguished over time, correlating to decreased cMET expression. Interestingly, we observed a rare population (∼1–2%) of cells with sustained cMET immunoreactivities, suggesting the possibility of retaining or transitioning to peptidergic profiles (Figure 2C). In comparison, when we employed a protocol to pattern into non-peptidergic DRG progenitors (Pettingill et al., 2019), we found RUNX1 upregulation and cMET downregulation, without the expression of peptidergic markers (Figure S2).

### Generation of TRPV1/TRPA1 non-peptidergic nociceptors from isolated placodes

We then characterized the identity of TG nociceptors matured from the RUNX1+/cMET- population of isolated placodes. Expression of markers typical of peripheral sensory neurons, such as PERIPHERIN, ISL1, BRN3A, TUJ1, and SYNAPTOPHYSIN could be confirmed (Figures 2A–C). Despite universal TRPV1 immunoreactivities and common expression of TRPA1, CGRP expression was mostly absent (Figure 2D). Non-peptidergic maker RET downstream of RUNX1 was expressed instead. As ion channels such as TRP family members are responsible for specialized stimulus detection *in vivo* (Levine and Alessandri-Haber, 2007; Usoskin et al., 2015a), we tested calcium influx in non-peptidergic TG nociceptors with selective agonists.

**Figure 3:**
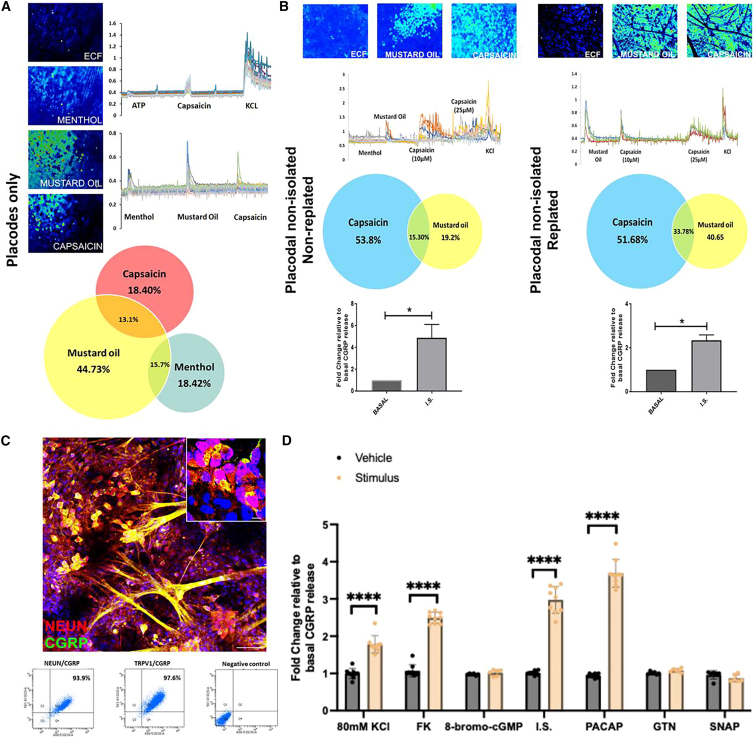
Presence of non-placodal cells re-directs neuronal cell fate specification, confirmed by differential functional competencies (A) Calcium imaging demonstrates that TG-placodal isolated nociceptors respond robustly to TRPA1 agonist mustard oil followed by high dose of TRPV1-agonist capsaicin giving them a non-peptidergic identity (*n* = 67 cells). (B) Calcium imaging demonstrates that TG cultures matured in presence of non-placodal cells with (left, *n* = 35 cells) and without (right, *n* = 54 cells) replating both generated nociceptors responsive to low dose capsaicin. (Bottom) CGRP ELISA demonstrated significant increase in CGRP release in response to I.S. (*n* = 3 independent replicates per stimulation, fold change quantified relative to basal release per stimulation, *p* < 0.05, one-way ANOVA). (C) Immunocytochemistry and analysis by flow cytometry for placodal non-isolated replated cultures (SBAD-03-01) demonstrates co-localization of mature neuronal marker NEUN with peptidergic marker CGRP, further validating their nociceptive identity, scale bars, 5 μm, inset scale bars, 50 μm. (D) CGRP release quantified by ELISA upon stimulation with inflammatory soup (I.S.), 80 mM KCl, FK, 1 μM PACAP-38, but not cGMP agonists (SNAP, GTN, 8-bromo-cGMP) (*n* = 9 independent replicates per stimulation, fold change quantified relative to basal release per stimulation, *p* < 0.05, one-way ANOVA). Basal release reflects the vehicle control.

The amplitude and number of cells with responses selectively driven by P2RX3 (10 μM ATP), TRPM8 (100 nM menthol), TRPA1 (250 μM mustard oil), and TRPV1 (25 μM capsaicin) were normalized against that of KCl (Figure 3, *n* = 67 cells). Many active neurons belonged to mustard oil-responsive TRPA1+ category (44.73%), followed by menthol-responsive TRPM8+ (18.42%) and capsaicin-responsive TRPV1+ (18.40%) categories. 13.1% of total responders were responsive to both capsaicin and mustard oil while 15.7% of total responders were reactive to both menthol and mustard oil. We found very few cells responded to ATP, or those reactive to both capsaicin and menthol (Figure 3). The lack of ATP response is consistent with rodent TG neurons, where most Ib4 non-peptidergic neurons do not express ATP-receptor P2rx3, unlike DRG Ib4 non-peptidergic neurons which do express P2rx3 (Ambalavanar et al., 2005). Therefore, we conclude the neurons matured from isolated placodes are consistent with a non-peptidergic TG profile.

### Generation of functionally competent *in vitro*-derived TG capsaicin-responsive peptidergic nociceptors

To profile the time course of CGRP expression during nociceptor development, we generated a *CALCA* promoter-driven GFP reporter line by targeting the *AAVS1* locus in human chromosome 19. Knock-in at the intron one of *PPP1R12C* gene was achieved using zinc finger nuclease (ZNF)-mediated obligate ligation-gated recombination (ObLiGaRe) (Maresca et al., 2013) (Figure 2E; Figures S3 and S4). Consistent with *CGRP* transcript quantification, GFP reporter was robustly present up to day 15 of differentiation and extinguished shortly after placode isolation (Figure 2F). However, *CALCA*-driven GFP signal was retained if the entire culture continued maturing without separating the placodes from the basal layer cells, with and without replating (Figures 1A and 2F).

We thereby hypothesized that the presence of non-placodal cells could modulate peptidergic commitment. Immunofluorescence identified co-expression of TRPV1 and CGRP in placodal neurons without physical separation from the basal layer cells, with and without subculturing (Figure 2D, CGRP-GFP reported line co-stained with CGRP antibody). Without replating, CGRP+ cultures were responsive to capsaicin (53.8% at 10 μM and 23% at 25 μM, Figure 3, *n* = 54 cells). Some neurons (19.2%) also responded to mustard oil, with 15.3% of total neurons responsive to both. A similar number of cells were responsive to capsaicin after passaging with non-placodal cells (51.68%, Figure 3, *n* = 35 cells). Interestingly, we found a greater population of cells reactive to mustard oil (40.65%), lifting the proportion of double responsive cells to 33.78%. Neither culture produced menthol-responsive cells.

We proceeded further functional characterization by replating single-cell extractions of both placodes and basal layers after Accutase digestion, as cultures without subculturing grew over-confluent due to the expansion of non-neuronal cells. Similarly, we could isolate single neurons from non-neuronal basal layer for flow cytometry analysis at day 55, of which 97.6% were TRPV1/CGRP double positive and 93.9% were NEUN/CGRP double positive (Figure 3B). We can conclude that our protocol generates a highly pure culture of hiPSC-derived peptidergic nociceptors.

We examined the electrophysiological properties of TG peptidergic nociceptors via whole-cell patch clamp recordings. Trains of action potentials were elicited in response to depolarizing current injection in current-clamp recordings. On some occasions spontaneous synaptic activity could be observed (Figure S5). Voltage-clamp recordings were made to further demonstrate that iPSC-derived neurons express the repertoire of voltage-gated ion channels characteristic of functional neurons. All recorded cells (*n* = 15) showed fast voltage activated inward currents followed by slow outward currents, consistent with sodium and potassium currents, respectively (Figure S5). Inward currents were blocked by 1 μM tetrodotoxin (TTX), a sodium voltage-gated channel blocker and TTX-resistant currents were also observed. (Figure S5). Although TTX-resistant responses were observed in several recordings, the proportion of TTX-resistant cells was not formally quantified.

### *In vitro* modulation of CGRP release of hiPSC-derived TG peptidergic nociceptors by migraine provocants and migraine treatments

Baseline CGRP secretion could be detected in TG peptidergic nociceptors, which could be further stimulated by known agonists such as inflammatory soup composed of ATP, bradykinin, PGE_2_, histamine, and noradrenaline (Maingret et al., 2008). Similar to primary rodent TG cultures (Basbaum et al., 2009; Durham and Russo, 1999), inflammatory soup increased secretion of CGRP by 3-fold and 5-fold in whole cultures with and without replating compared to corresponding baselines (Figure 3C, *n* = 3 per line). Other known positive stimulants including FK, PACAP-38 (Vollesen and Ashina, 2017), and depolarization signal KCl also significantly increased CGRP release, whereas nitroglycerin (GTN), sodium nitroprusside (SNAP), and 8-bromo-cGMP did not.

**Figure 4:**
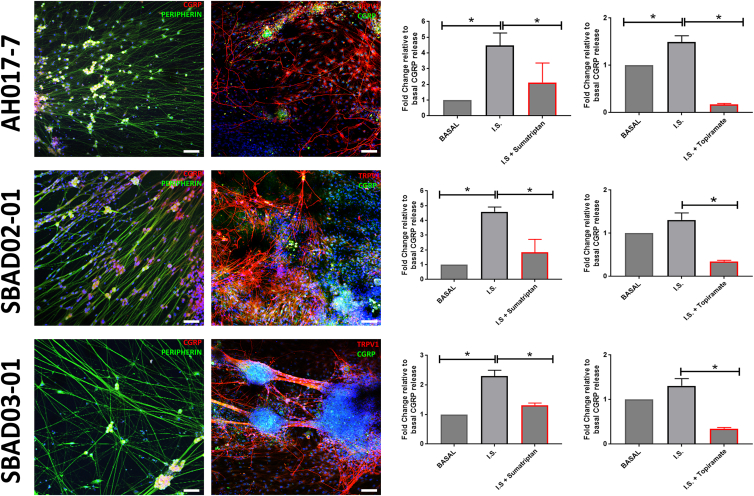
Efficient reproducibility of peptidergic differentiation protocol in AH017-7, SBAD03-01 and SBAD02-01 human iPSCs lines (Left) Immunocytochemistry reveals peptidergic nociceptor identity of the TG neurons as confirmed by the expression of CGRP, PERIPHERIN and TRPV1. (Right) Drug-inhibition assay on I.S.-stimulated CGRP release quantified by ELISA (*n* = 3 independent replicates per iPSC line per stimulation, fold change quantified relative to basal release per stimulation, *p* < 0.05, one-way ANOVA).

Many of the current migraine drugs may mediate their effects through altering CGRP release. As 5-HT1D receptor (site of action of sumatriptan) was expressed in our peptidergic cultures (Figure S6), we also tested whether an acute administration of common migraine treatment could alter CGRP release. Indeed, inflammatory soup-evoked CGRP release was significantly inhibited in the presence of sumatriptan or topiramate in multiple wild-type lines (*n* = 3 separate dishes per iPSC line, Figure 4).

### Single-cell transcriptomic analysis reveals human TG-like characteristics of the *in vitro*-derived TG peptidergic model

**Figure 5:**
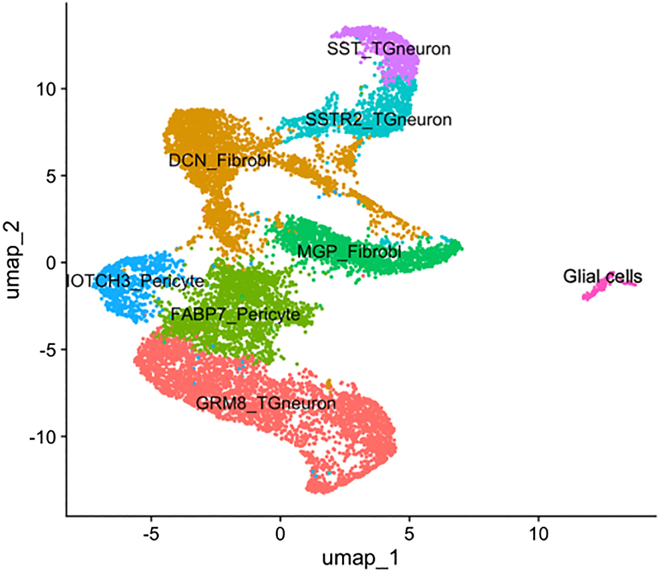
UMAP visualization of scRNA-seq data from replated TG neuronal cultures UMAP projection of single-cell RNA sequencing data showing transcriptionally distinct cell clusters within the replated TG neuronal culture. Each dot represents an individual cell, colored and labeled by annotated cell identity based on marker gene expression. Multiple neuronal subtypes are evident, including GRM8-, SST-, and SSTR2-expressing neurons. Non-neuronal populations include fibroblast-like cells (DCN^+^, MGP^+^), pericyte-like cells (NOTCH3^+^, FABP7^+^), and a small, distinct glial cell cluster.

To confirm the identity of iPSC-derived replated TG nociceptor cultures, we undertook single-cell transcriptomics using the 10X genomics platform. We identified discrete cellular populations within the culture, with uniform manifold approximation and projection (UMAP) analysis demonstrating segregation of neuronal and non-neuronal lineages. Neuronal cells segregated into multiple transcriptionally distinct subtypes, reflecting neuronal heterogeneity and subtype specification. Non-neuronal populations included fibroblast-like cells and pericyte-like cells, with a small but distinct glial population also present (Figure 5).

We constructed a cell type-specific gene expression space through principal component analysis of different human brain cell types and then projected iPSC-derived nociceptor single cells from the control samples onto this space. We observed that all iPSC-derived nociceptor cells clustered much closer to the external TG/DRG samples than to the other cell types (Flegel et al., 2015; LaPaglia et al., 2018) (Figure S7). Moreover, while these iPSC-derived nociceptors could be formed into distinct clusters based upon their transcriptomic variation, once projected onto the cell type-specific gene expression space, all iPSC-derived nociceptor cells did not separate by the identified clusters suggesting cell clusters were not defined by varying cell identity (Figure S7). For this reason and because of the very low and noisy expression of marker genes at the single cell level, we merged the reads from all cells to create a pseudo-bulk sample that we further compared with external TG/DRG samples based on selected marker genes (Figure S7). The peptidergic identity of our replated TG nociceptors was confirmed by the expression of *TRPV1*, *CGRP* (*CALCA*), *TAC1*, and *PACAP* similar to as observed in postmortem human TG samples as previously described (LaPaglia et al., 2018). Despite TRPA1-agonist induced calcium flux was observed in these cultures, we did not detect the expression of *TRPA1* in the analysis, which perhaps could be attributed to low number of reads detected for this gene. These nociceptors also expressed sensory neuronal markers, including *ISL1*; *BRN3A*; markers indicative of pain vasculature, such as *MTDH*, *TGFBR2*, and *JAG1*; synaptic markers *SYT4* and *SYNPR* and importantly; glial marker *GLUL*; and Schwann cell marker *MPZ* (Figure S7) which could perhaps be the key cell types facilitating the maturation of these replated TG cultures to release CGRP.

### Astrocytic co-culture of placodal-isolated TG cultures promotes peptidergic nociceptive features

**Figure 6:**
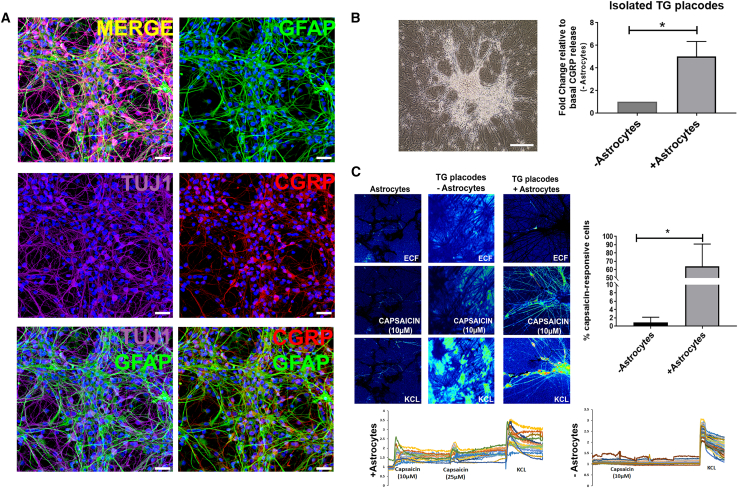
Co-culture of isolated TG placodes with astrocytes induces peptidergic nociceptive fate (A) Immunocytochemistry reveals peptidergic identity of TG placodes-astrocyte co-cultures indicated by GFAP-expressing astrocytes and neurons expressing both CGRP and TUJ1, scale bars, 25 μM. (B) (Left) Fura-2-AM excitation images showing changes in calcium flux in neuron-astrocyte co-cultures in response to stimuli, scale bars, 10 μM. (Right) Functional analysis via calcium imaging suggests increased sensitivity to capsaicin in placode-astrocyte co-cultures similar to replated cultures (*n* = 3 independent replicates, *p* < 0.05, one-way ANOVA). (C) Astrocyte co-cultures facilitate basal CGRP release compared to TG placodes cultured in the absence of astrocytes, (*n* = 3 independent replicates per line per condition, fold change quantified relative to basal release, *p* < 0.05, one-way ANOVA).

The single-cell transcriptomic analysis on the replated peptidergic TG nociceptor cultures revealed the expression of glial markers, and it is widely recognized that astrocytes help support neuronal survival and maturation (Allen et al., 2012). Although glial cells represented only a small fraction of the populations identified by single-cell RNA sequencing (scRNA-seq), sensory neurons normally develop in close association with glia, including satellite glial cells and Schwann cells. We therefore tested the hypothesis that glial co-culture could support placodal differentiation into peptidergic sensory neurons. An additional rationale was that co-culturing placodes with rat glial cells would allow tighter control over culture composition by introducing a defined number of glial cells while minimizing the presence of other cell types observed in replated cultures. We observed that isolated TG placodal nociceptors co-cultured with primary rat astrocytes now expressed CGRP as confirmed by CGRP/TUJ1 co-expression in 66.78% of the cells whilst GFAP expressing astrocytes showed no CGRP expression (Figure 6A). These co-cultures also exhibited 5-fold increase in basal CGRP release, compared to those cultured in the absence of astrocytes (Figure 6B), although we did not observe inflammatory soup evoked release. Analysis by calcium imaging further confirmed functional competence in these neuron-astrocyte co-cultures, where approximately 60% of the nociceptors responded to 10 μM capsaicin and no such response was seen in placodal-isolated neurons cultured in the absence of astrocytes (Figure 6C). Hence, these data suggests that the neuronal-astrocyte interactions aids in fate determination with the induction of CGRP expression.

## Discussion

The diversification of nociceptors into sub-classes is a multi-step process likely involving early patterning of neuronal progenitors followed by postmitotic differentiation towards a mature nociceptor subtype-specific phenotype, governed by both intrinsic signals which determine their differentiation potential and the surrounding niche that influence the population size of nociceptor subtypes (Barres et al., 2015). Peptidergic neurons gain Runx1^+^/TrkA^+^/cMet^+^ identity whereas the non-peptidergic acquire Runx1^+^/TrkA^+^/Ret^+^ expression profile. As development progresses and functional maturation occurs through the perinatal and postnatal period, the peptidergic nociceptors lose Runx1, giving them a cMet^+^/TrkA^+^/Cgrp^+^ identity and the non-peptidergic neurons continue to express Ret, a receptor for a glial-derived neurotrophic factor (GDNF), thereby exhibiting a Runx1^+^/Ret^+^ expression profile (Gascon et al., 2010; Liu and Ma, 2011b). Hence, the mechanism controlling nociceptor fate specification is attributed to intrinsic determinants such as functional expression of cMet, as well as to extrinsic factors including the balance of NGF, HGF, and GDNF signaling which may be required for the persistence or extinction of Runx1 expression (Gascon et al., 2010).

We found in TG differentiation, that following placodal induction, the sensory neurons markers ISL1 and BRN3A were expressed, along with the nociceptor marker TRKA. We observed expression of RUNX1 at early stages which then increased along with expression of RET confirming a non-peptidergic nociceptor identity. A recently described comprehensive unbiased RNA-seq study of 622 mouse neurons (Usoskin et al., 2015b) has brought to light the plethora of nociceptor subtypes that exist, at least based upon transcriptomic identity. The functional relevance of these many subtypes remains to be established. The TG placodal-isolated nociceptors comprised a large population of TRPA1^+^/TRPV1^-^/TRKA^+^/RET^+^/CGRP^−^ cells which are similar to the NP1 category from the RNA-seq classification and a minority population of TRPA1^+^/TRPV1^+^/TRKA^+^/RET^+^/CGRP^−^ cells which match the non-peptidergic NP2/NP3 category. Interestingly, we found that unlike neural crest-derived non-peptidergic neurons from human iPSC, our TG placode derived cells did not respond to ATP, normally mediated by P2RX3 receptors in sensory neurons. However an examination of rat TG sensory neurons indicated only a minority of Ib4^+^ non-peptidergic neurons express P2rx3, whilst in DRG Ib4^+^ sensory neurons, 60%–70% expressed P2rx3 (Ambalavanar et al., 2005). Our findings are therefore consistent with a TG lineage of non-peptidergic nociceptors.

Peptidergic nociceptors are an important nociceptor subtype in health and disease including inflammatory and neuropathic pain and migraine (Lukacs et al., 2017), and a human *in vitro* model of these nociceptors would help advance disease modeling and therapy development for many pain-related disorders. We now report for the first time, the highly efficient generation of human iPSC-derived functional model of TG peptidergic nociceptors. Almost all the peptidergic nociceptors, expressed TRPV1, aligning with the peptidergic PEP1 category of mouse neurons (Usoskin et al., 2015b) and were responsive to capsaicin. TRPV1^+^ peptidergic nociceptors are considered to mediate noxious thermal pain behaviors and consistently no cells responded to menthol, indicating an absence of functional cold sensor, TRPM8. PEP1 (CGRP^+^) and TRPM8 being two separate populations is fitting with the human TG sequencing data (Yang et al., 2022).

A substantial proportion of peptidergic nociceptors expressed TRPA1 (Cavanaugh et al., 2009; Le Pichon and Chesler, 2014; Ro et al., 2009). In mouse models, Trpv1 is expressed widely expressed in DRG neurons initially and then becomes restricted to peptidergic nociceptors. Distribution of Trpv1 in rat (Price and Flores, 2007) and TRPV1 in human (Jung et al., 2023; Shiers et al., 2020, 2021; Tavares-Ferreira et al., 2022; Yang et al., 2022) nociceptors appear to be broader, blurring the non-peptidergic and peptidergic boundary. We found that our iPSC differentiated nociceptors expressed functional TRPV1 in both non-peptidergic and peptidergic subtypes although the different nociceptor subpopulations had differential responsiveness to capsaicin (Demartini et al., 2017; Durham and Masterson, 2013; Hoffmann et al., 2013). The non-peptidergic nociceptors expressing TRPV1 only responded to 25 μM capsaicin whilst peptidergic nociceptors were sensitive to 10 μM capsaicin, as has been reported for mouse TG peptidergic nociceptors (Hoffmann et al., 2013). Similarly, we have also found that TRPA1 can be expressed in both peptidergic and non-peptidergic nociceptors. We cannot exclude the possibility that the high concentration of mustard oil used here induced partial TRPA1-independent effects. At elevated levels, it may affect membrane integrity or engage additional molecular targets, potentially contributing to the observed responses. This limitation should be considered when interpreting specificity.

The *CALCA*-GFP iPSC reporter line revealed that CGRP gene expression increases early in the TG differentiation protocol and is clearly present at the cranial placode stage before becoming rapidly extinguished after placodal isolation. The decline in CGRP expression corresponded to the decrease in cMET expression in these cultures, consistent with the previous studies indicating the importance of cMet in the production of peptidergic nociceptors (Gascon et al., 2010). Although some studies have indicated a low overall level of CGRP in sensory neurons during the pre-natal period, earlier studies have demonstrated that TG neurons innervating the cerebrovasculature are enriched for CGRP expression (O’connor and Van Der Kooy, 1988). TG neurons innervate peripheral targets by E14-15 and are expressing CGRP a few days later (Tsai et al., 1989). Hence our *in vitro* TG neurons may be analogous to those TG neurons that supply to the cerebral blood vessels, with early expression of CGRP. Our single cell transcriptomic analysis also confirmed the similarity of TG peptidergic nociceptors with the previously published data on human TG (LaPaglia et al., 2018). While there were apparent distinct sub-clusters present when examining the iPSC nociceptors alone, once projected onto the cell type-specific gene expression space, cell clusters were not defined by varying cell identity probably due to the very low and noisy expression of nociceptor marker genes at the single cell level.

Given the early expression of cMET and CGRP, it seemed that with the appropriate signals, the TG cultures could adopt a peptidergic fate. This was confirmed when we either did not isolate the placode or replated the whole culture after the placode had formed at a lower density and found the overwhelming majority of neurons were peptidergic nociceptors. This suggests that other cells in the culture outside of the cranial placode are providing an extrinsic signal for peptidergic fate specification perhaps akin to target derived signals *in vivo*. Remarkably we found that astrocytes may be able to provide much of the extrinsic signaling required to generate peptidergic nociceptors from the cranial placode. Astrocytes are widely recognized to support neuronal function and promote neuronal survival and maturation by releasing trophic factors including NGF and BDNF during development (Allen and Barres, 2009; Allen et al., 2012; Seth and Koul, 2008). We also demonstrated CGRP expression in 65.8% of cells the TG placodal isolated nociceptors which otherwise exhibit only non-peptidergic features in the absence of astrocytes. While we observed a basal release of CGRP with astrocyte co-culture, we were unable to evoke release with inflammatory soup, suggesting that other factors may be required for evoked release.

In rodent models of migraine, application of inflammatory soup to the dura leads to activation of TG neurons, increased CGRP release into the jugular vein (Hoffmann et al., 2009) and increased CGRP expression in the TG (Lukács et al., 2015). In our culture, exposure of the TG peptidergic nociceptors to inflammatory soup potently led to increased release of CGRP. Moreover, we found that the migraine provocant PACAP-38 but not GTN, was able to evoke CGRP release. We speculate that CGRP release in our *in vitro* nociceptors is mediated via cAMP pathway since we also observed CGRP release with FK but not with cGMP-pathway agonists, including SNAP, GTN, and 8-bromo-cGMP (De Mey and Vanhoutte, 2014; Miller and Megson, 2007) (Figure 3F). Sumatriptan is a highly effective acute migraine therapeutic drug and was developed to target the 5-HT1 receptor subtypes (5-HT_1B_, 5-HT_1D_, and 5-HT_1F_) on blood vessels to induce vasoconstriction (Ahn and Basbaum, 2005). The vascular changes in migraine are now not considered primary and instead there is considerable focus on CGRP, based upon increased CGRP levels during migraine attacks and the success of clinical trials of CGRP receptor antagonists and more recently the monoclonal antibodies against CGRP or the CGRP receptor (Goadsby et al., 2017; Oliveira et al., 2024; Silberstein et al., 2017). The 5-HT_1B/1D/1F_ receptors have been shown to localize on the presynaptic terminals of TG neurons (Ahn and Basbaum, 2005), and sumatriptan has been shown to directly inhibit the stimulated but not the basal CGRP release from cultured rat TG neurons (Durham and Russo, 1999). Importantly, we found that sumatriptan was able to prevent the stimulated release of CGRP but did not affect basal CGRP release. We also observed inhibitory effect of topiramate on stimulated CGRP release. The ability of migraine provocants to induce CGRP release and migraine treatments to reduce CGRP release supports the importance of CGRP in migraine pathophysiology and also illustrates the potential value of iPSC-peptidergic nociceptors as a disease model and basis of a phenotypic screening assay.

Future work will investigate the interaction of maturing nociceptors with the adjacent cells including glia to decipher CGRP release mechanism, as it is clearly apparent that replating placodes with the surrounding niche promotes generation of functional TG peptidergic nociceptors, whilst isolating the placodes in the absence of astrocytes leads to the induction of non-peptidergic nociceptors. The knowledge gained in this study paves way to generate a more *in vivo*-like ganglionic composition of non-peptidergic and peptidergic nociceptors to identify specific signaling cues and crosstalk between the different sensory neuronal subtypes. This will enable translational studies to improve drug discovery in pain disorders that continue to face significant unmet medical need.

## Resource availability

### Lead contact

Further inquiries and resource requests should be directed to Dr. Galbha Duggal (galbha.duggal@oxfordstemtech.com).

### Materials availability

iPSC lines generated in this study will be made available upon request, but we may require a payment and/or a completed materials transfer agreement if there is potential for commercial application.

### Data and code availability

Single-cell RNA-seq data have been deposited at GEO at GEO: GSE331403 and are publicly available as of the date of publication. All data reported in this paper will be shared by the lead contact upon request. This paper does not report original code.

## Acknowledgments

The research leading to these results has received support from the 10.13039/501100010767Innovative Medicines Initiative Joint Undertaking under grant agreement no. 115439, resources of which are composed of financial contribution from the European Union’s Seventh Framework Programme (FP7/2007–2013) and 10.13039/100013322EFPIA companies’ in-kind contribution. This research has also been supported by the 10.13039/501100013373NIHR Oxford Biomedical Research Centre. This publication reflects only the author’s views and neither the IMI JU nor EFPIA nor the European Commission are liable for any use that may be made of the information contained therein. We would also like to thank Prof. Dave Bennett, Dr. Alex Clark, and Dr. Gregory Weir for their help with calcium imaging set-up and analysis as well as critical reading of the manuscript.

## Author contributions

Conceptualization, G.D. and M.Z.C.; methodology, G.D., S.C., S.K.G, M.M., and R.H.; software, C.W.; validation, G.D., S.C., C.F.H., P.P., T.L., D.C., K.A., and X.L.; formal analysis, G.D., V.V., and C.W.; investigation, G.D. and S.C.; resources, T.L., R.B., H.S., and Z.C.; data curation, V.V. and C.W.; writing – original draft, G.D.; writing – review and editing, G.D., X.L., and M.Z.C.; visualization, G.D., T.L., and M.Z.C.; supervision, M.Z.C.; project administration, G.D., S.C., and M.Z.C; funding acquisition, M.Z.C.

## Declaration of interests

The authors declare no competing interests.

## STAR★Methods

### Key resources table

**Table:** 

REAGENT or RESOURCE	SOURCE	IDENTIFIER
**Antibodies**		

Rabbit anti-PAX6	Biolegend	Cat# 901301; RRID: AB_2565003
Goat anti-SIX1	SCBT	Cat# sc-9709; RRID: AB_641021
Rabbit anti-CASPASE3	Abcam	Cat# ab13847; RRID: AB_443014
Goat anti-RUNX1	SCBT	Cat# sc-8564
Rabbit anti-cMET	Abcam	Cat# ab51067; RRID: AB_880695
Rabbit anti-ISLET-1	Abcam	Cat# ab20670; RRID: AB_881306
Goat anti-PERIPHERIN	SCBT	Cat# sc-7604; RRID: AB_2171328
Rabbit anti-BRN3A	Abcam	Cat# ab81213; RRID: AB_1640222
Mouse anti-TUJ1	Biolegend	Cat# 801201; RRID: AB_2313773
Rabbit anti-SYNAPTOPHYSIN	Abcam	Cat# ab14692; RRID: AB_301417
Rabbit anti-TRPV1	Alomone Labs	Cat# ACC-030; RRID: AB_2313819
Rabbit anti-5HT_1D_	Alomone Labs	Cat# ASR-023
Mouse anti-CGRP	Abcam	Cat# ab81887; RRID: AB_1658411
Rabbit anti-TRKA	Alomone Labs	Cat# ANT-018; RRID: AB_10658910
Rabbit anti-RET	Alomone Labs	Cat# ANT-025; RRID:AB_2341013
Rabbit anti-NEUN	Milipore	Cat# ABN78; RRID: AB_10807945
Chicken anti-GFAP	Abcam	Cat# ab4674; RRID: AB_304558

**Chemicals, peptides, and recombinant proteins**		

Y-27632	Abcam	Cat# ab120129
NOGGIN	R&D Systems	Cat# 6057-NG
SB431542	Tocris	Cat# 1614
BDNF	Peprotech	Cat# 450-02
Ascorbic Acid	Sigma	Cat# A5960
Forskolin	Sigma	Cat# F6886
Laminin	Sigma	Cat# L2020
PACAP-38	Sigma	Cat# A1439
SNAP	Sigma	Cat# N3398
8-bromo-cGMP	Sigma	Cat# B1381
Potassium Chloride	Sigma	Cat# P9541
Prostaglandin E2	Sigma	Cat# P0409
Histamine	Sigma	Cat# H7125
Bradykinin	Sigma	Cat# B3259
Noradrenaline	Sigma	Cat# A7257
ATP	Sigma	Cat# A2383
Menthol	Sigma	Cat# M2772
Allyl isothiocyanate	Sigma	Cat# 377430
Capsaicin	Sigma	Cat# M2028
Sumatriptan Succinate	Sigma	Cat# S1198
Topiramate	Sigma	Cat# T0575
Potassium Chloride	Sigma	Cat# P4504
Sodium Chloride	Sigma	Cat# S9625
HEPES	Sigma	Cat# 54457
D-Glucose	Sigma	Cat# G7021
Calcium Chloride	Sigma	Cat# 21115
Magnesium Chloride	Sigma	Cat# 63069
FURA-2	Thermofisher Scientific	Cat# 11524766
Pluronic Acid	Thermofisher Scientific	Cat# 10767854

**Critical commercial assays**		

CGRP (human) ELISA kit	Bertin Pharma	Cat# A05481

**Deposited Data**		

Raw and Analysed Data	This paper	GEO: GSE331403

**Experimental models: Cell lines**		

Human iPSC lines	StemBANCC Consortium	SBAD-03-01 SBAD-02-01
Human iPSC line	University of Oxford	AH017-7
Rat Primary Cortical Astrocytes	Thermofisher Scientific	Cat# N7745100

**Oligonucleotides**		

Primer (SA-T2A-Puro amplification) Forward: 5′-ACGCGTCACTCTCGAGGGAGAGGGCAGAG GAAGTCTTCTAAC-3′	This paper	N/A
Primer (SA-T2A-Puro amplification) Reverse: 5′-ACGCGTCACTCTCGAGCCATAGAGCCCAC CGCATCCCC-3′	This paper	N/A

**Recombinant DNA**		

pUC57CGRP-GFP targeting vector	This paper	N/A
pUC57CGRP-GFP-SA-T2A-Puro plasmid	This paper	N/A
pUC57CALCA-GFP-T2A-Puro targeting vector	This paper	N/A
Zinc Finger Nuclease (ZFN) plasmid targeting AAVS1 locus	This paper	N/A
Splice acceptor–T2A–Puromycin cassette	This paper	N/A

**Software and algorithms**		

10x Genomics Cell Ranger v7.2	Cell Ranger 7.2 | Official 10x Genomics Support	https://www.10xgenomics.com/support/software/cell-ranger/7.2
Seurat R package v2.4	Butler et al., 2018	https://satijalab.org/seurat/archive/v2.4/pbmc3k_tutorial

### Experimental model and study participant details

#### Human iPSC culture

Human iPSC lines (AH017-7, SBAD-03-01, SBAD-02-01) were derived from fibroblasts of healthy individuals after informed consent, using CytoTune-iPS Sendai Reprogramming Kit (Life Technologies). The iPSCs were derived as part of the IMI/EU sponsored StemBANCC consortium. Cell lines were cultured in feeder-free system on Matrigel™ (Corning) and were fed with mTESR1™ media (STEMCELL Technologies). Cultures were passaged every 5-7 days or when 75-95% confluent, using EDTA (Life Technologies) and split at 1:4 ratio with medium changed every day.

### Method details

#### Generation of CGRP-GFP targeting plasmid vector

To construct pUC57CGRP-GFP-T2A-Puro for expression of GFP reporter, CGRP promoter sequence -2053 to +91 (Genebank ID: AC090835.7, bases 45451-47599) was synthesized by ThermoFisher Scientific. The Splice acceptor-T2A-puro was amplified by PCR from a plasmid using primer pair 5′ACGCGTCACTCTCGAGGGAGAGGGCAGAGGAAGTCTT CTAAC3′ and 5′-ACGCGTCACTCTCGAGCCATAGAGCCCACCGCATCCCC-3′ (XhoI site underlined). The PCR product was ligated with the XhoI sites of the vector pUC57CGRP-GFP. The resulting plasmid pUC57CGRP-GFP-SA-T2A-Puro, consisted of Zinc Finger site (ZFN) for AAVS1 locus.

#### Transfection of iPSCs with targeting vector and Zinc Finger Nuclease (ZFN) plasmids

StemBANCC iPSC line SBAD03-01 was selected for targeting as it was derived from a healthy donor, showed a stable karyotype and efficiently differentiated towards all three lineages (data not shown). SBAD03-01 cells were harvested using TrypLE (Gibco) and 300.000 cells/well of a 12 well plate were co-transfected with two plasmids: one expressing Zinc Finger Nucleases (ZFN) and the other containing the CALCA promoter, fluorescent reporter and a drug-resistance cassette using Lipofectamine LTX (ThermoFisher Scientific). We used a targeting plasmid vector, pUC57CALCA-GFP-T2A-Puro, in which the last GFP coding codon is fused in frame with a T2A sequence followed by puromycin. Both ends of the insert are loxP-flanked (floxed) in order to remove it if needed. Transfected cells were passaged once confluent and selection with puromycin (0.2μg/ml) was performed after 24 hours. When puromycin-resistant colonies expanded, single cell cloning was performed through serial dilution in 384 well plates to identify pure clones.

#### Genotyping by Junction PCR and ddPCR

Junction PCR and ddPCR analysis identified multiple correctly targeted clones without random transgene integration from a total of 300+ puromycin-resistant clones screened. Junction PCR was performed using primers pairs from both 5’ end and 3’ end of the insert to identify clones with insert at correct location. DNA from clones was isolated using Modified Gitschier Buffer (MGB) and PCR was performed using Phusion high fidelity Taq pol (ThermoFisher Scientific). For ddPCR analysis, droplets were generated using QX200 droplet generator and FAM/Hex labelled probes. PCR products were then analyzed by QX200 droplet reader and QuantaSoft software.

The targeting efficiency (7%) was comparable to the efficiencies observed with TALENs and CRISPR/Cas9 using similar targeting strategies (Hockemeyer et al., 2009, 2011). CGRP-GFP clones 1-4, 5-17 & 5-40 were identified as single copy insertion and 1-8, 1-14 & 1-38 were identified as two copy insertion based on ddPCR data. For our study, CGRP-GFP clone C1-4 was employed.

#### Differentiation of human iPSCs towards TG and DRG nociceptors

Directed differentiation of iPSCs towards TG nociceptors was performed as previously described (Dincer et al., 2013). Briefly, cells were lifted as single cells in Accutase (Stem Cell technologies) and plated at 60,000 cells/cm^2^ in the presence of 10μM Y-27632 (Abcam) and 10ng/ml bFGF (Peprotech) in MEF-conditioned media. Upon 90-95% confluency, neural induction was initiated using 250ng/ml Noggin (R&D Systems) and 10μM SB431542 (Tocris) for 3 days in basal media composed of DMEM-F12, Glutamax, 20% Knockout Serum Replacement (Life Technologies). From day 4, cells were cultured only in the presence of 10μM SB431542 and from day 5 onwards, N2B27 media (B27 supplement without vitamin A, Life Technologies) was added in increments of 25%, 50%, 75% every 2 days, for 13-17 days leading to the formation of placodes. Next, three different maturation strategies were adopted. First, only the placodes were manually isolated and plated on Matrigel™ up to days 55-65 in maturation media composed of N2 supplement and B27 without Vitamin A Supplement (Thermofisher Scientific), 20ng/ml BDNF (Peprotech), 0.2mM Ascorbic acid (Sigma) and 2μM Forskolin (Sigma). Second, the TG placodes were not isolated and the maturation medium was added from day 15 onwards. Third, day 15 cultures were lifted using Accutase (placodes and the surrounding cells) and they were replated on Matrigel™ at 200.000/cm^2^ cell density in maturation medium followed by downstream analysis day 55 onwards. DRG nociceptors were differentiated from iPSCs using previously widely published protocol (Chambers et al., 2012; Pettingill et al., 2019).

#### Immunocytochemistry

Samples were fixed in 4% paraformaldehyde (Sigma) for 20 minutes at room temperature (RT). They were then permeabilized for eight minutes in 0.1% Triton X-100 (Sigma) diluted in 1X phosphate buffered saline (PBS), washed in 1X PBS for five minutes and blocked for one hour in 1% BSA, 0.05% Tween-20 in 1X PBS at RT. Subsequently, cells were incubated in primary antibodies (diluted in blocking solution) at 4°C overnight, washed three times in 1X PBS for five minutes and treated with secondary antibodies (diluted in blocking solution) for one hour at RT. Next, samples were washed again three time in 1X PBS for five minutes, incubated with DAPI for 10 minutes and mounted on glass slides using Dako Mounting Fluorescence medium (Dako). Primary antibodies used were rabbit anti-PAX6 (1:200, Biolegend), goat anti-SIX1 (1:100, SCBT), rabbit anti-CASPASE3 (1:200, Abcam), goat anti-RUNX1 (1:200, SCBT), rabbit anti-cMET (1:200, Abcam), goat anti-PERIPHERIN (1:100, SCBT), rabbit anti-ISLET1 (1:200, Abcam), rabbit anti-BRN3A (1:100, Abcam), mouse anti-TUJ1 (1:1000, Biolegend), rabbit anti-SYNAPTOPHYSIN (1:100, Abcam), rabbit anti-TRPV1 (1:100, Alomone Labs), rabbit anti-5HT_1D_ (1:100, Alomone labs), mouse anti-CGRP (1:100, Abcam), rabbit anti-TRKA (1:100, Alomone Labs), rabbit-anti RET (1:200, Alomone Labs), rabbit anti-NEUN (1:500, Milipore), chicken anti-GFAP (Abcam) and mouse anti-TUJ1 (Abcam). Secondary antibodies used were donkey anti-goat Alexa488, donkey anti-rabbit Alexa594, goat anti-rabbit Alexa488, goat anti-mouse Alexa594, goat anti-mouse Alexa488 and goat anti-rabbit Alexa594. All secondary antibodies were from ThermoFisher Scientific and were used at 1:500 dilution. All images were acquired by confocal microscopy using Zeiss 880 inverted microscope (Zeiss).

#### Quantitative Real-time PCR

Total RNA was isolated using TRIzol and RNeasy Mini kit (Qiagen). Reverse transcription was performed on 300ng RNA per sample using SuperScript First-Strand Synthesis kit (Thermofisher Scientific). The cDNA was diluted 1:5 for each sample and qRT-PCR was performed using SybrGreen PCR Master Mix (ThermoFisher Scientific) and Applied Biosystems 7500 Fast Real-Time PCR machine. Housekeeping genes included *GAPDH* and *βACTIN* and genes of interest used were *RUNX1*, *cMET* and *CGRP*. All samples were analysed as biological triplicates (n = 3 for each sample). Negative controls included both water (no cDNA) and no RT (RNA) samples. The CT values were normalized against housekeeping genes and fold change in gene expression was analyzed using ΔΔCT method. Statistical significance was determined using one way-ANOVA and p<0.05 was considered significant.

#### CGRP ELISA

Medium was collected from the different culture conditions, centrifuged at 200g for 5 minutes and supernatants were frozen at -80°C in multiple aliquots to avoid freeze-thaw cycles. Briefly, basal media, 80mM KCl stimulated-media (4-hour exposure) and inflammatory soup^21^, 2μM FK, 1μM PACAP-38, 1mM SNAP, 1μM 8-bromo-cGMP-stimulated media and 1μM GTN (Sigma) were collected following 24 hours exposure (n = 3 separate dishes per iPSC line for each treatment). For drug-inhibition assay, media was collected from the same dishes that were first pre-treated with 10μM sumatriptan or 100μM topiramate (Sigma) for one hour, followed by exposure to I.S. supplemented with either of the drugs (n = 3 separate dishes per iPSC line per treatment). CGRP ELISA was conducted using Human CGRP Enzyme Immunoassay Kit as per manufacturer’s instruction (Bertin Pharmacy/Bioquote). Plates were read on a Wallac Victor 1420 Multilabel counter at 405nm for 0.1s and data was analyzed on GraphPad Prism 6 software. Statistical significance was determined using one way-ANOVA and p<0.05 was considered significant.

#### Calcium imaging

Mature nociceptors from all the three culture methods (placode isolation, no isolation and replating) were loaded with 2μM Fura2-AM and 80μM Pluronic acid (ThermoFisher Scientific) and incubated at 37°C for one hour. They were then washed 1X PBS and fed with in-house formulated extracellular fluid (ECF, composed of 145mM NaCl, 5mM KCl, 10mM HEPES, 10mM D-Glucose, 2mM CaCl_2_, 1mM MgCl_2_, pH 7.4). For imaging, excitation filters BP340/30 and BP387/15 were used to capture images every 2 seconds and the ratiometric 340/380 calculations were performed by subtracting the background. Baseline imaging was performed for 30 seconds in ECF followed by 30 second exposure to the agonists with five-minute ECF wash between each agonist perfusion. ATP (10μM) was applied for 30 seconds, followed by capsaicin (10μM, 25μM) and finally KCl (50mM) or the cells were exposed sequentially to menthol (100nM), mustard oil (250μM, Belinskaia et al., 2023), capsaicin (10μM, 25μM) and KCl (50mM).

#### Flow cytometry

Placodal non-isolated replated cultures from non-GFP iPSC line (SBAD03-01) were gently washed with PBS to isolate neurons from non-neuronal cell layer. The isolated neurons were treated with accutase for 10 minutes, centrifuged at 400g for 5 minutes and the cell pellet was re-suspended in 2% PFA (Alfa Aesar) diluted in FACS buffer (1% FBS, 10μg/ml human serum albumin and 0.01% Azide made up in 1X PBS) for 10 minutes at RT. They were then permeabilized in 1ml ice cold 100% methanol for 30 minutes at -20°C or stored up to several weeks for later use. Next, approximately 500,000 cells were harvested, spun at 2000rpm for three minutes, followed by wash in FACS buffer and split into two for each antibody combination. Cells were incubated in primary antibody for 1 hour at RT, washed in FACS buffer, incubated with secondary antibodies for 45 minutes at RT and washed and re-suspended in FACS buffer for analysis. Primary and secondary antibodies used were the same as previously described for immunocytochemistry including NEUN, TRPV1 and CGRP. Isotype controls were used as negative controls.

#### Electrophysiology

Human iPSC-derived nociceptors were plated directly onto the Ibidi dishes (Thistle Scientific) for functional characterization. Individual ibidis were placed in a recording chamber mounted onto the stage of an upright microscope. For targeted whole-cell recordings, we used a 40x water immersion objective and IR-DIC optics. Cells were continuously superfused with oxygenated aCSF (95% O_2_/5% CO_2_) containing 130mm NaCl, 25mm NaHCO_3_, 2.5mm KCl, 1.25mm NaH_2_PO_4_, 2mm CaCl_2_, 2mm MgCl_2_ and 10mm glucose. Patch-clamp electrodes (4–7 MΩ) were filled with an intracellular solution containing 120mm K-gluconate, 10mm KCl, 10mm HEPES, 4mm MgATP, 0.3mm NaGTP and 10mm Na-phosphocreatine. Recordings were obtained using a Multiclamp 700B amplifier and digitized at 10–20 kHz using Digidata 1550 acquisition board.

#### Single-cell RNA sequencing

iPSC-TG nociceptors were washed in PBS with 0.04% BSA and re-suspended at a concentration of ∼1500cells/μl before capturing single cells in droplets on Chromium 10x Genomics platform. Library generation for 10x Genomics v2 chemistry was performed following the Chromium Single Cell 3ʹ Reagents Kits User Guide: CG00052 Rev B. Quantification of cDNA was performed using Qubit dsDNA HS Assay Kit (Life Technologies Q32851). Quantification of library construction was performed using Qubit dsDNA HS Assay Kit (Life Technologies Q32851) and high-sensitivity DNA tapestation (Agilent. 5067-5584). Libraries were sequenced on Illumina HiSeq4000 platform to achieve an average of 42,000 reads per cell.

#### Alignment, barcode assignment and UMI counting

We used the pipelines “mkfastq” and “count” from the Cell Ranger Single-Cell Software Suite (https://support.10xgenomics.com/single-cell-gene-expression/software/pipelines/) to demultiplex raw base call files into FASTQ files, and to perform alignment, barcode counting and UMI counting. Multiple sequencing runs (two HiSeq4000 lanes) of the same library were combined by the “count” pipeline. Barcodes and UMIs were filtered using default settings to generate gene-barcode matrix.

#### Clustering analysis

We used Seurat R package (Butler et al., 2018) for QC filtering and data analysis. Only genes with at least one UMI count detected in at least one cell were used. Only cells with less than 10% of sequenced mitochondrial genes and more than 200 expressed genes were kept. This left 12257 cells and 25541 genes for analyses, with approximately ∼2,000 genes expressed in each cell. UMI normalization was performed using the function “NormalizeData” that consisted in dividing UMI counts by the total expression in each cell, followed by multiplication with a scale factor (10,000 by default), and by log-transforming the UMI counts. The top 2,000 most variable genes were identified using the function “FindVariableGenes” with parameters x.low.cutoff = 0.0125, x.high.cutoff = 3, y.cutoff = 0.3. We regressed out cell-cell depth variation in gene expression using the number of detected UMIs with the function “ScaleData”. The function “FindClusters” was used to perform a graph-based clustering approach on the normalised and scaled dataset with parameters reduction.type = “pca”, dims.use = 1:5, resolution = 0.3. To confirm the identified clusters, we used an unsupervised clustering method for single cell RNAseq data called SC3 (Kiselev et al., 2017). We found that five of the eight clusters identified by the two methods shared more than 50% of the cells while three were less robust.

#### Cell type gene expression PCA space

We merged RNA-Seq data from purified human brain cell types including neurons, astrocytes, oligodendrocytes and endothelial cells available at http://www.brainrnaseq.org (Zhang et al., 2016), from isolated microglia during surgery of cortical regions in patients with epilepsy (Gosselin et al., 2017), from human trigeminal ganglia (TG) and dorsal root ganglia (DRG) (Flegel et al., 2015) and from human TG (LaPaglia et al., 2018) . PCA (scaled and centered) was performed on the merged dataset and the internal 10x single cells were projected onto this space using the “predict” R function.

### Quantification and statistical analysis

Information regarding number of differentiations, lines used, and statistical analyses can be found in the figure legends. All statistical tests were performed using GraphPad Prism 10. Statistical analysis for cell culture experiments across multiple differentiations were performed using one-way ANOVA with p < 0.05 considered significant, unless otherwise stated. Data are plotted as mean ± SD. For single cell RNAseq, Seurat R package was used for QC filtering and data analysis.

## Supplemental information

Document S1. Figures S1–S7 and Tables S1 and S2

Document S2. Article plus supplemental information
