# Supplementary material for: A highly efficient method to differentiate CGRP-expressing peptidergic nociceptors from human induced pluripotent stem cells
Source: Stem Cell Reports. 2026 Jun 25;21(7):102971. doi: 10.1016/j.stemcr.2026.102971 (PMC13385448; doi:10.1016/j.stemcr.2026.102971)
Supplement: Document S1. Figures S1–S7 and Tables S1 and S2 [file mmc1.pdf]

Stem Cell Reports, Volume *21*

Supplemental Information

A highly efficient method to differentiate CGRP-expressing peptidergic nociceptors from human induced pluripotent stem cells

Galbha Duggal, Xinyu Li, Philippa Pettingill, Tatjana Lalic, Shailesh Kumar Gupta, Christine Flodgaard Høgsbro, Viola Volpato, Caleb Webber, Rory Bowden, Despoina Charou, Kanisa Arunasalam, Marcello Maresca, Ryan Hicks, Satyan Chintawar, and M. Zameel Cader

Supplementary Figure 1

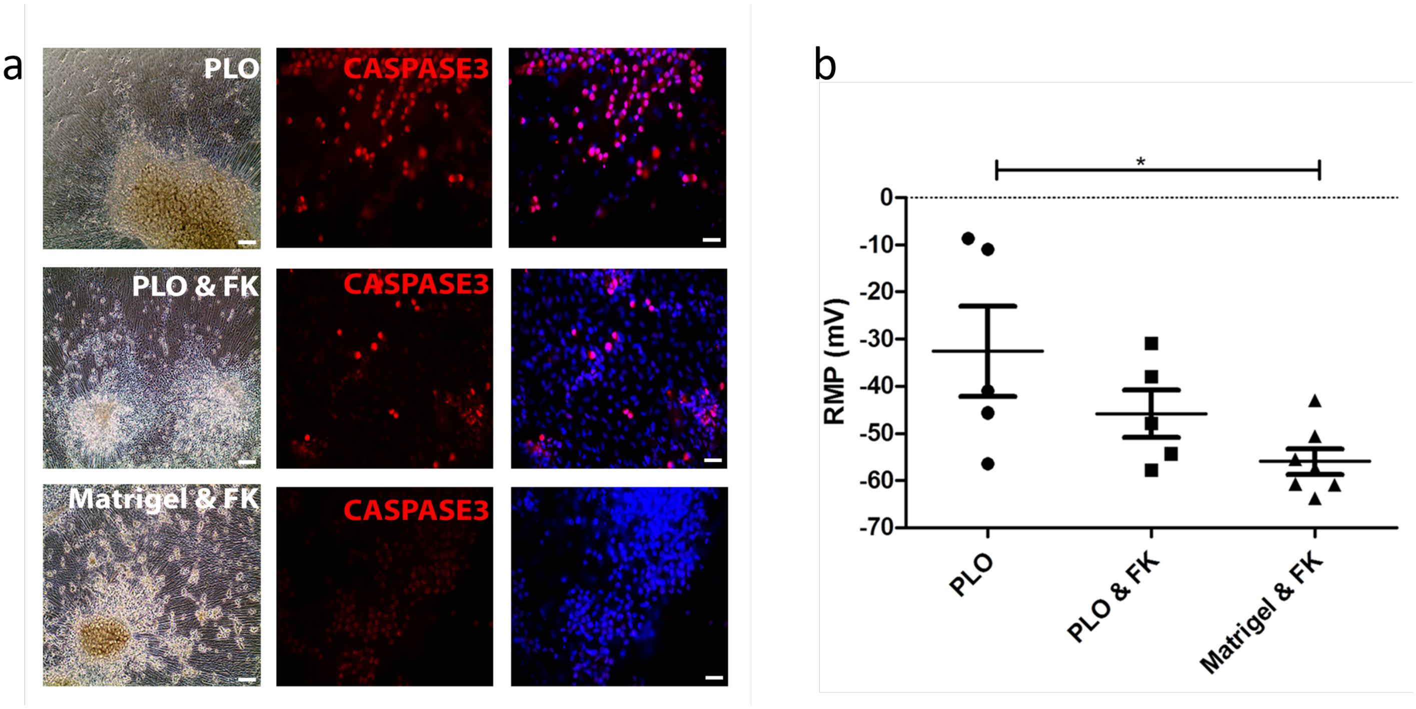

Supplementary Figure 2

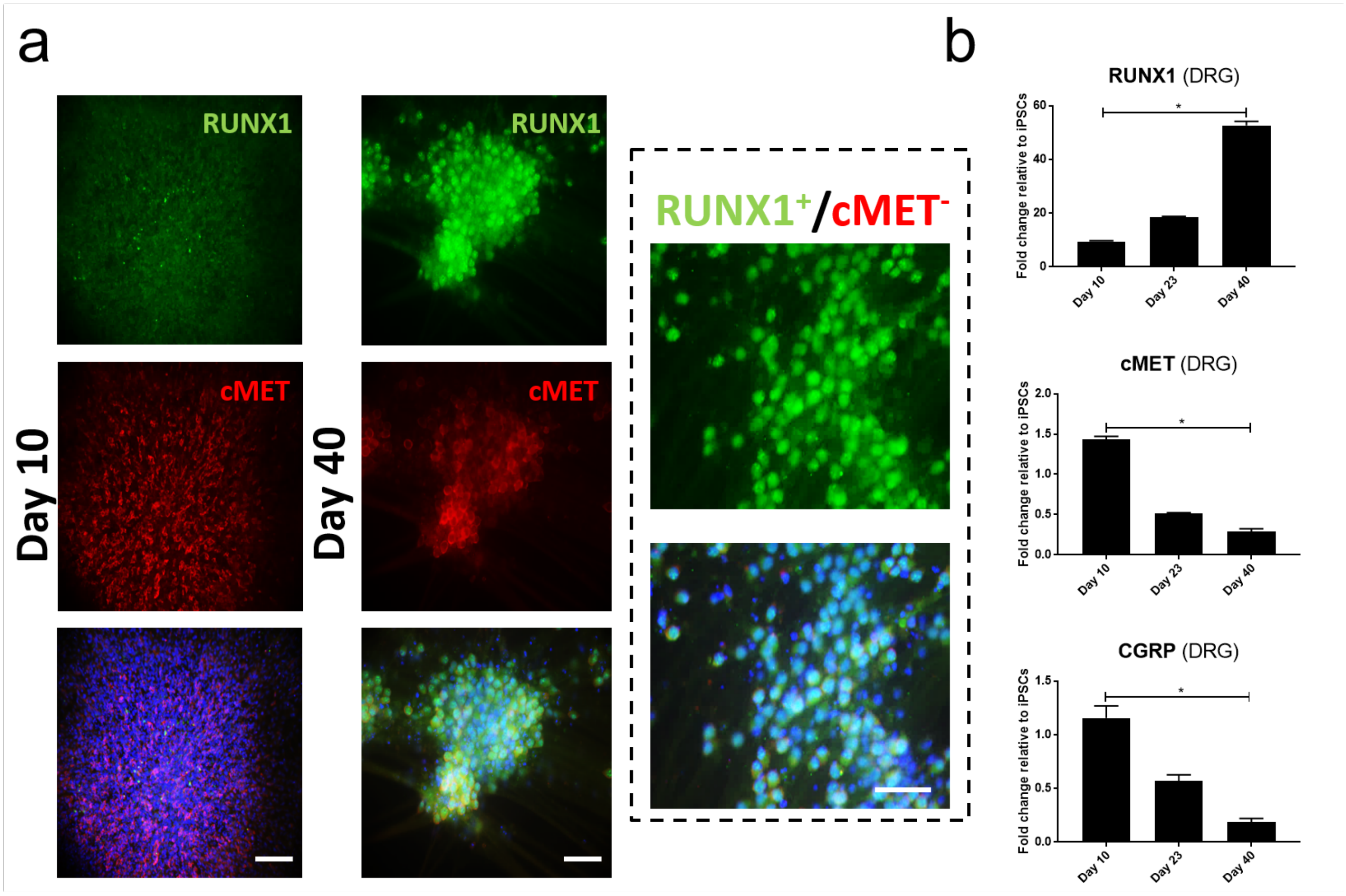

Supplementary Figure 3

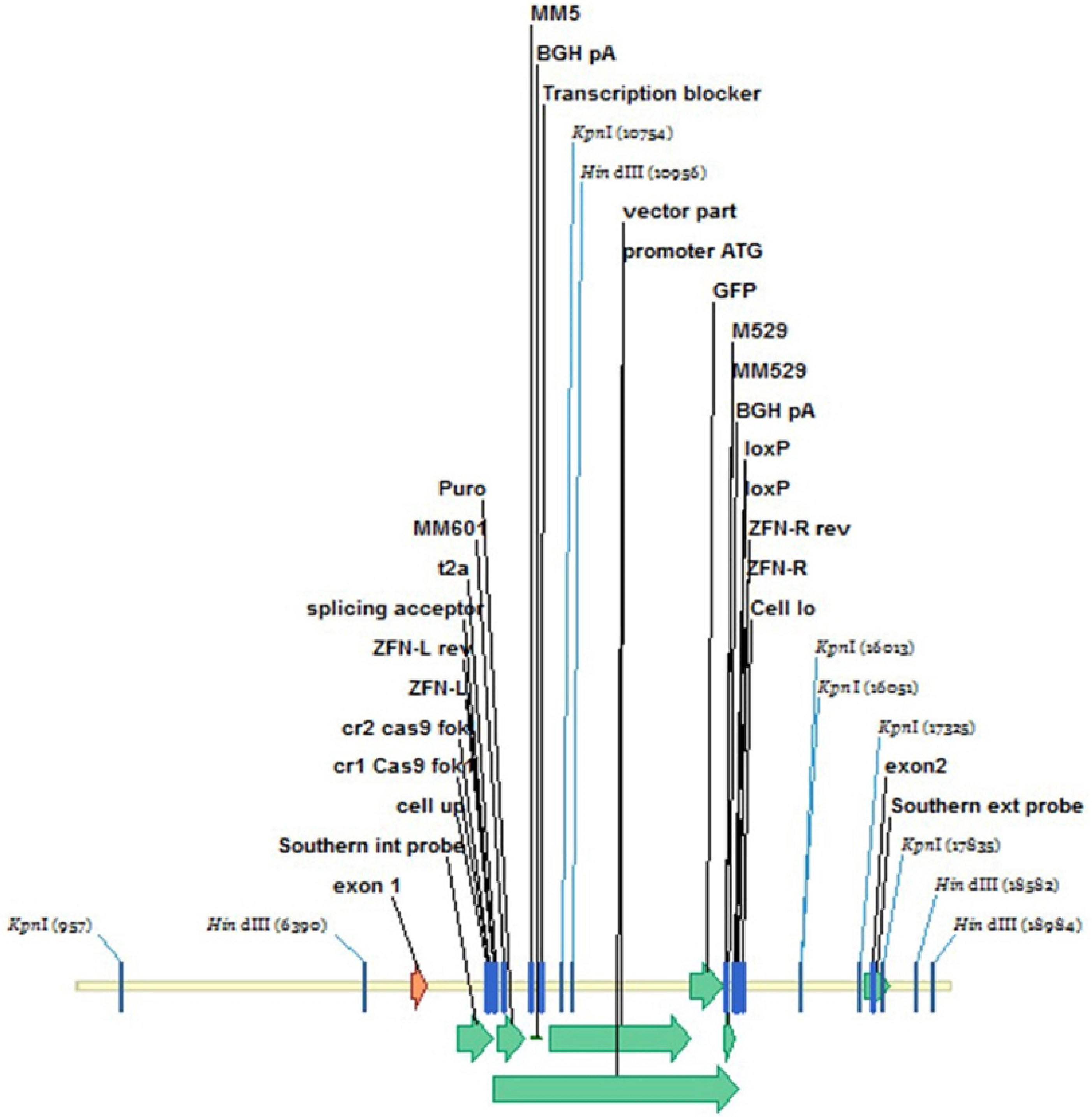

hAAVS1:CGRP-GFP
(19335bp)

Supplementary Figure 4

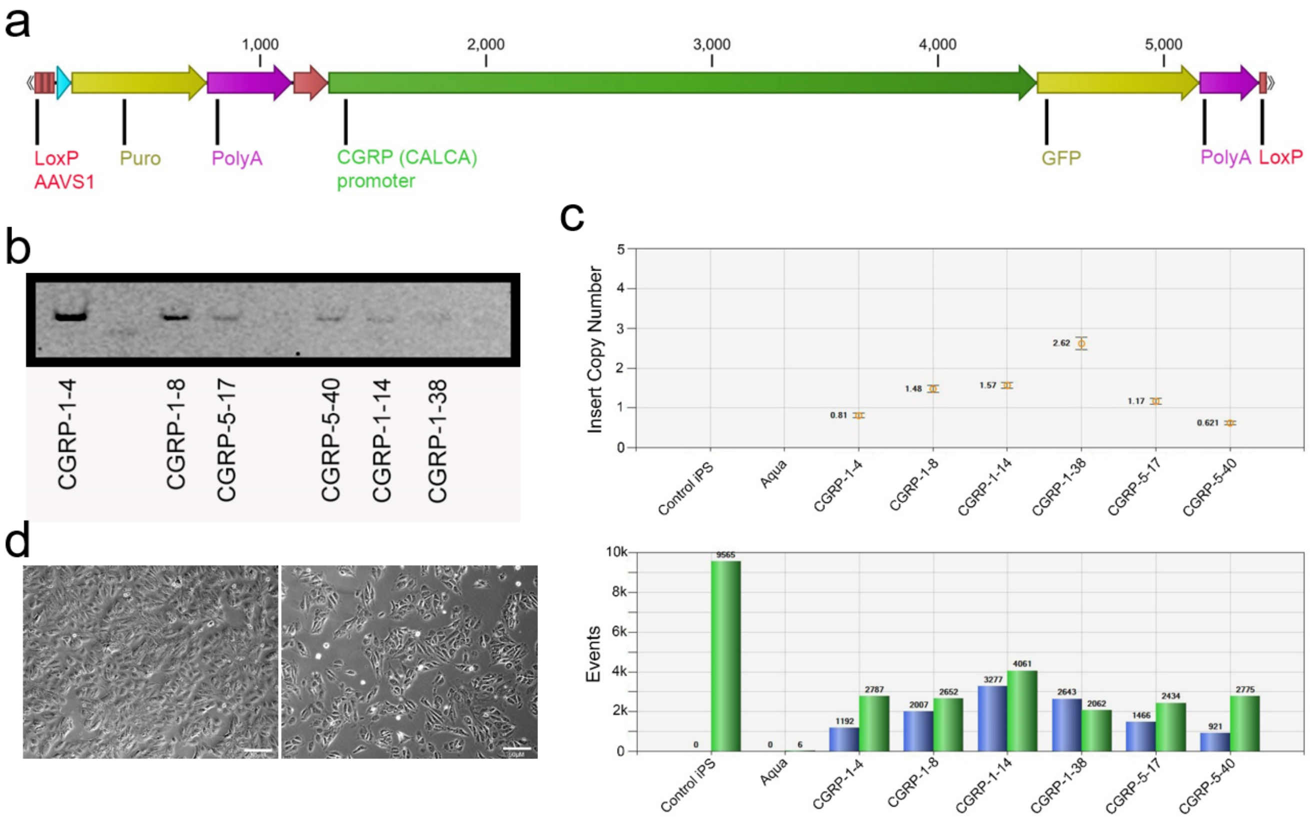

Supplementary Figure 5

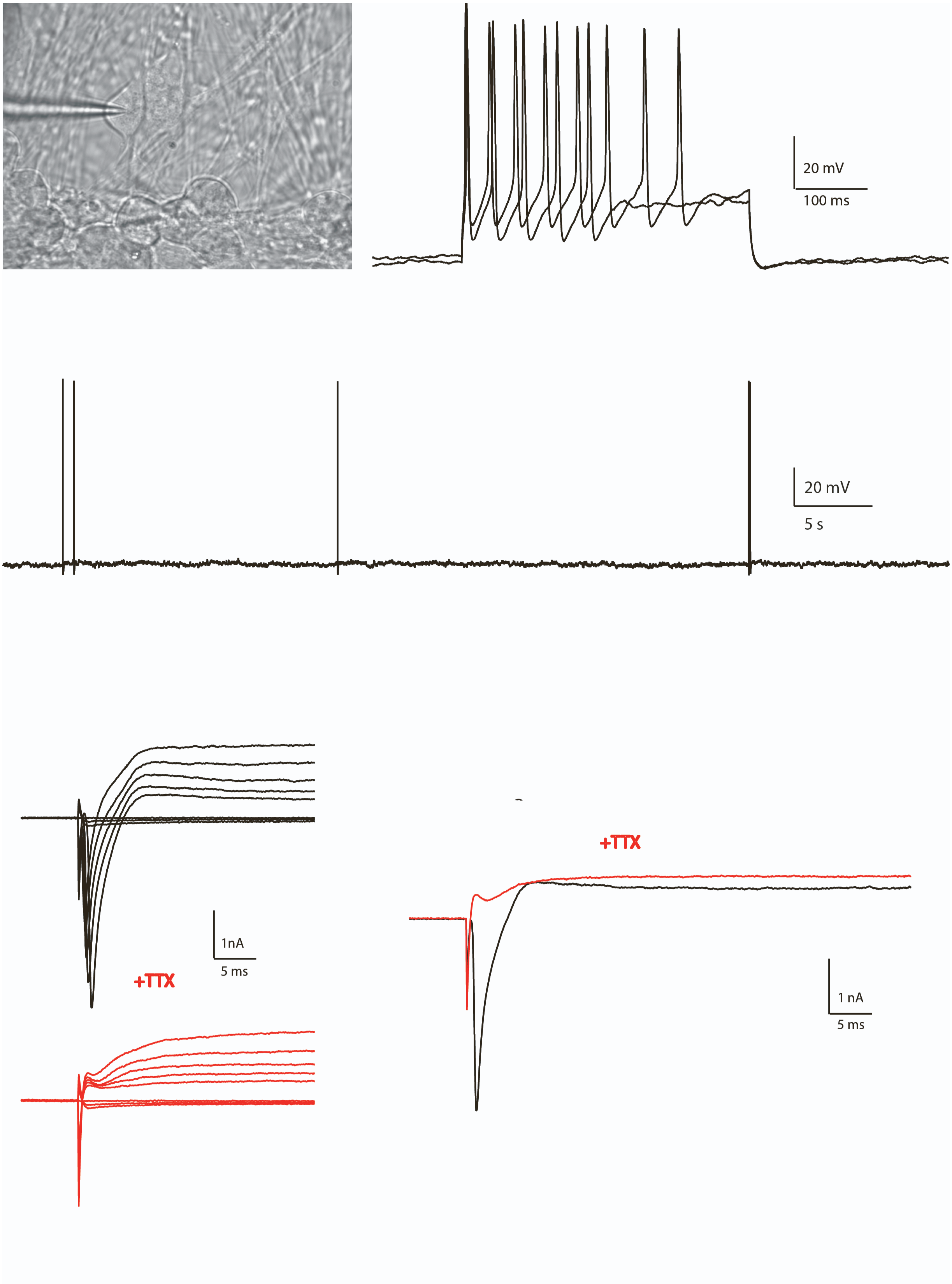

Supplementary Figure 6

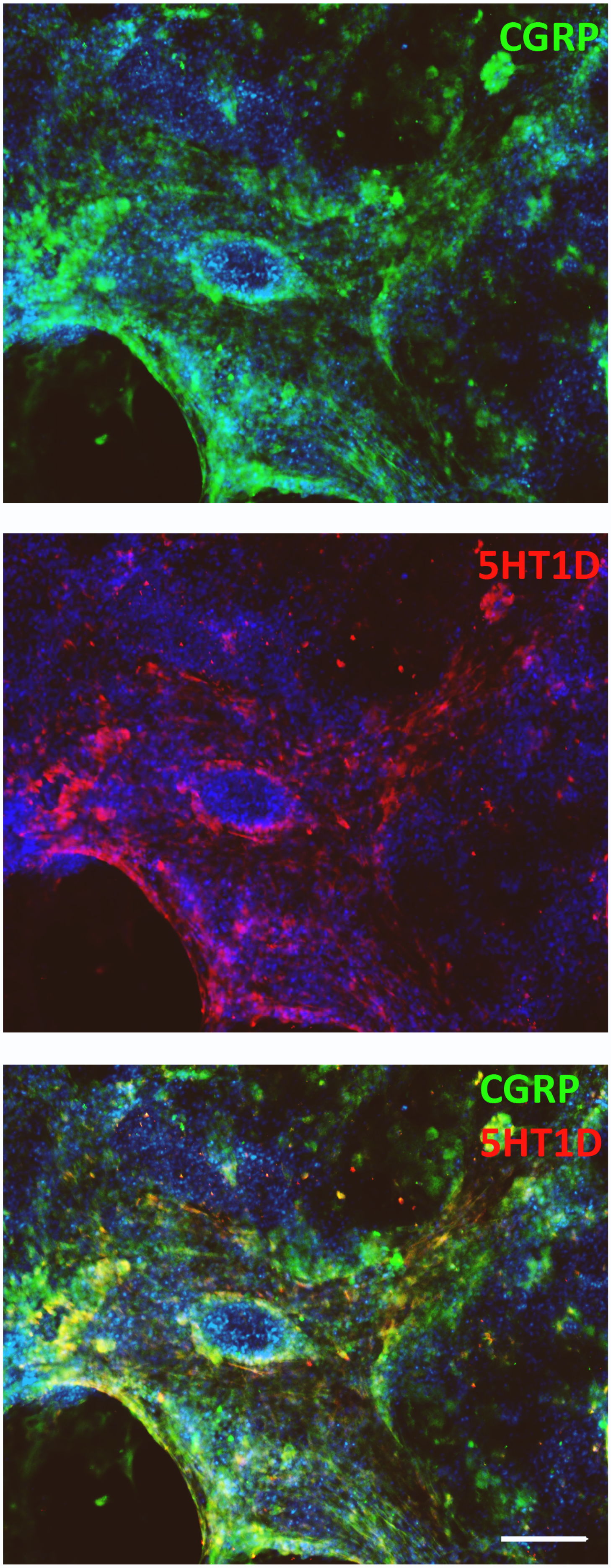

Supplementary Figure 7

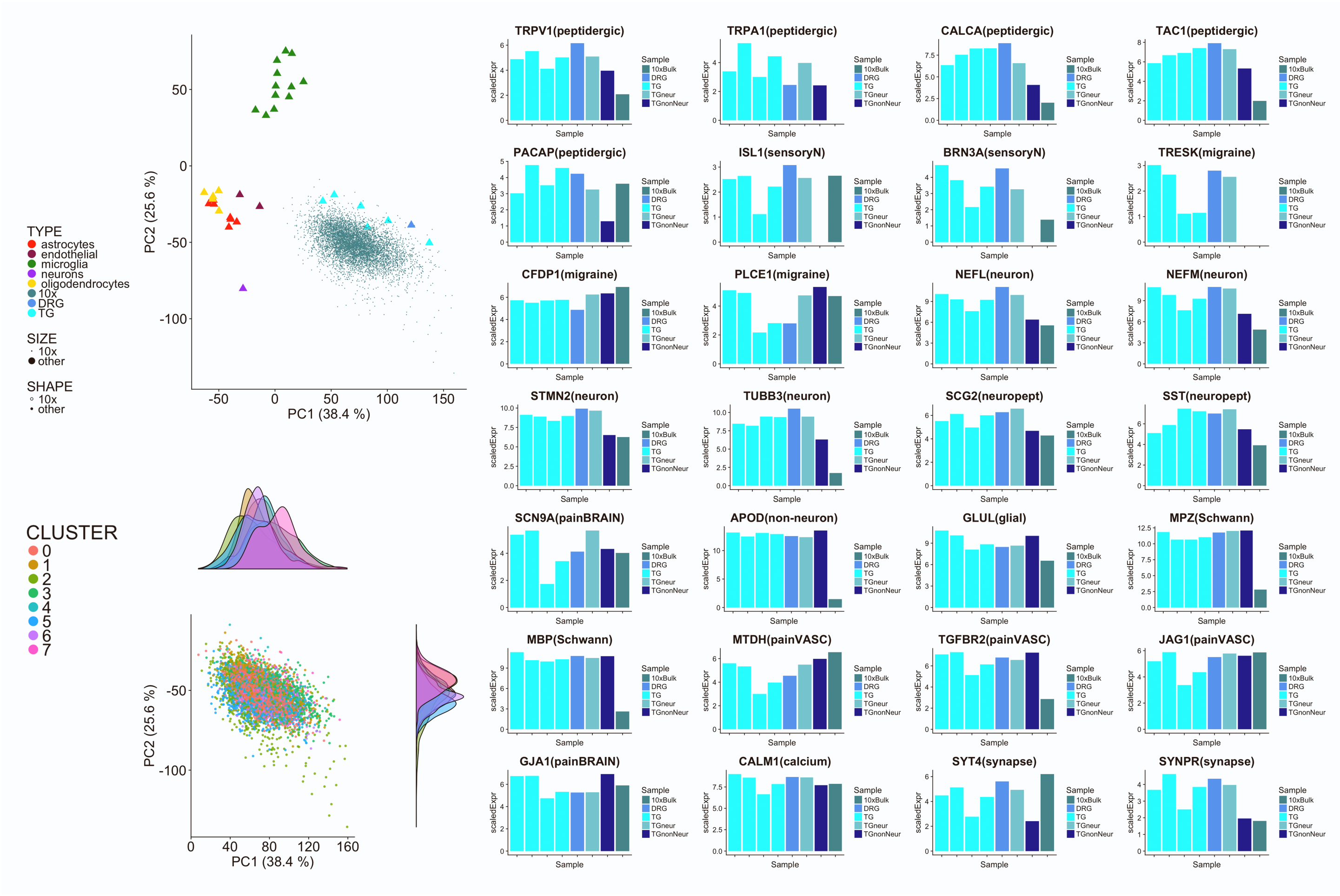

Supplementary Figures

Supplementary Figure 1: Optimization of TG differentiation protocol. TG nociceptors generated in the presence of matrigel and FK combination **a)** reduces cell death as shown by CASPASE-3 expression and **b)** are functional and have a physiological resting membrane potential of -60mV (n = 12 cells per iPSC line, p<0.05, one-way ANOVA).

Supplementary Figure 2: RUNX1 and cMET expression profiling during DRG nociceptor differentiation to identify peptidergic and non-peptidergic population. a) RUNX1 expression increases while cMET expression decreases as differentiation progresses, scale bar 10μm. Dashed box reveals small population that has $RUNX1^+/cMET^-$ non-peptidergic identity at day 40, scale bar 25μm. **b)** Gene expression profiling by qRT-PCR demonstrates significant increase in non-peptidergic *RUNX1* marker while peptidergic markers *cMET* and *CGRP* are significantly downregulated (n = 3 independent replicates, p<0.05, one-way ANOVA).

Supplementary Figure 3: Schematic showing the detail map of CGRP-GFP-T2A-Puro insert. This was used in the generation of CGRP-GFP reporter line.

Supplementary Figure 4: ObLiGaRe Mediated Targeting of the AAVS1 Locus through drug selection. a) Schematics of the targeting strategy. The presence of the ZFNs and targeting plasmid vector, results in the insertion of pCGRP-GFP-T2A-Puro cassette at *AAVS1* locus in human chromosome 19. In the targeting schematics here, colored boxes are various segments of the insert that is flanked by LoxP sequence. **b)** The PCR primers (GFP-F + R) used for genotyping for identifying correctly targeted clones. Six of the correctly targeted clones (clone 1-8, 1-14, 1-38, 1-4, 5-17 & 5-40) were selected for further characterization & validation. **c)** ddPCR analysis indicating insert copy number. Internal puromycin probe was used for analyzing the clones, showed zero insert copy number for control iPSC, two copies for clones

1-8, 1-14 & 1-38 and one copy for clones 1-4, 5-17 & 5-40. Blue bar represents total events number for puromycin probe and the green bar represents total number of events for reference gene *AP3B1*. Aqua sample had no template DNA.

Supplementary Figure 5: Electrophysiological analysis of TG peptidergic nociceptors derived upon non-placodal isolation replating strategy. The somas of differentiated neurons were targeted by DIC for whole cell recordings as illustrated in the figure (n = 15 cells per iPSC line). Example traces of current clamp recordings of repetitive firing and occasional spontaneous activity in iPSC-derived nociceptors. Voltage clamp recordings show large inward Na currents that are TTX sensitive. Peptidergic neurons display repetitive firing in response to depolarization and spontaneous activity indicating functional neuron/maturation (left panel).

Supplementary Figure 6: Co-expression of $5HT_{1D}$ receptor and CGRP. Analysis by immunocytochemistry in non-placodal isolated replated TG peptidergic nociceptor cultures, scale bar 10μm.

Supplementary Figure 7: 10x single cell analysis confirms peptidergic-like sensory neuron identity for all cells. (Left panel, top) Cell type identity space built through PCA of external RNAseq brain cell types. When projected onto this space, iPSC-derived nociceptor single cells cluster near the external TG and DRG samples. (Left Panel, bottom) iPSC-derived nociceptor single cells projected on the same space do not separate by the cell clusters identified Seurat (see Methods). (Right panel) Gene expression levels for given genes within the combined single cells (pseudo-bulk) obtained by merging all reads from all single cells, and for external TG and DRG samples. Gene expression is scaled on housekeeping genes. The functional classification for each gene is reported within parentheses based on information derived from TG and DRG samples and from TG "neuron" and TG "non-neuron" (LaPaglia et al., 2017).
